# Low-dose intranasal dexmedetomidine premedication improves epidural labor analgesia onset and reduces procedural pain on epidural puncture: a prospective randomized double-blind clinical study

**DOI:** 10.1186/s12871-023-02146-5

**Published:** 2023-05-30

**Authors:** Hao Sun, Xiang Ma, Shengyou Wang, Zhenzhen Li, Yao Lu, Haijuan Zhu

**Affiliations:** 1 Department of Anesthesiology, the First Affiliated Hospital of Anhui Medical University, Hefei, 230022 China; 2 Department of Anesthesiology, Anhui Maternal and Child Health Care Hospital, Maternal and Child Health Care Hospital of Anhui Medical University, Hefei, 230001 China; 3 Department of Anesthesiology, the Third Affiliated Hospital of Anhui Medical University, Hefei, 230061 China

**Keywords:** Dexmedetomidine, Intranasal, Labor pain, Onset labor, Procedural pain

## Abstract

**Background:**

Epidural labor analgesia is a safe and effective method of pain management during labor with the drawbacks of delayed onset and maternal distress during epidural puncture. This study aimed to determine whether pretreatment with intranasal low-dose dexmedetomidine effectively shortens the onset of analgesia and reduces procedural pain.

**Methods:**

In this prospective, randomized double-blind trial, nulliparous patients were randomly assigned to either the intranasal dexmedetomidine group or the control group. The intranasal dexmedetomidine group received 0.5 μg/kg dexmedetomidine intranasally, and the control group received an equal volume of normal saline intranasally. Both groups were maintained with a programmed intermittent epidural bolus. The primary outcome was the onset time of analgesia and scores of pain related to the epidural puncture.

**Results:**

Seventy-nine patients were enrolled, and 60 completed the study and were included in the analysis. The time to achieve adequate analgesia was significantly shorter in the intranasal dexmedetomidine group than in the control group (hazard ratio = 2.069; 95% CI, 2.187 to 3.606; *P* = 0.010). The visual analogue scale pain scores during epidural puncture in the intranasal dexmedetomidine group were also significantly lower than those in the control group (2.0 (1.8–2.5) *vs.* 3.5 (3.3–4.5), *P* ≤ 0.001, Table 2). Pretreatment with intranasal dexmedetomidine before epidural labor analgesia was associated with improved visual analogue scale pain scores and Ramsay scores, less consumption of analgesics and higher maternal satisfaction (*P* < 0.05). No differences were observed for labor and neonatal outcomes or the incidence of adverse effects between the two groups.

**Conclusions:**

Pretreatment with intranasal dexmedetomidine before epidural labor analgesia yielded a faster onset of analgesia and decreased epidural puncture pain without increasing adverse effects. Pretreatment with intranasal dexmedetomidine may be a useful adjunct for the initiation of epidural analgesia, and further investigation should be encouraged to determine its utility more fully.

**Trial registration:**

This trial was prospectively registered at Chictr.org.cn on 29/05/2020 with the registration number ChiCTR2000033356 (http://www.chictr.org.cn/listbycreater.aspx).

## Background

Epidural analgesia (EA) has excellent clinical efficacy and is a preferred choice for pain relief during labor [1]. However, it has a few drawbacks, such as the delayed onset of analgesia and pain during epidural puncture, which often causes maternal fear and anxiety [2]. Dural puncture epidural (DPE) is a modification of the traditional continuous epidural infusion (CEI), which is thought to quicken the onset of analgesia [3]. However, a recent systematic review of five randomized controlled trials suggested that the efficacy of DPE has not been determined to date [4].

Dexmedetomidine (DEX) is a highly selective agonist of α2-adrenergic receptors that possesses sedative, anxiolytic and analgesic effects with low placental transfer and low respiratory depression, and it does not affect foetal physiologic status [5–7]. Due to these attractive factors, DEX was reported as an adjuvant for labor analgesia via epidural or intravenous access to prolong the analgesic duration, improve analgesic quality, deepen sedation, and reduce the use of analgesics such as opioids which have side effects, while so far no reports has shown that it quickens the onset of analgesia [8, 9]. In addition to the epidural or intravenous route, DEX has also been confirmed to be effective through intramuscular, oral, perineural and intranasal routes [10–13]. Intranasal dexmedetomidine (IND) (2.5 μg/kg) has been shown to be well tolerated, fast acting, safe and efficacious in patients undergoing radiation therapy who require light to moderate sedation and analgesia [14]. IND has also been reported to reduce procedural distress, aid postoperative analgesia, and relieve anxiety and fear in premedicated pediatric patients [11, 12]. Previous studies have shown that IND is rapidly and efficiently absorbed into the bloodstream via the nasal mucosa with less irritation than other sedative drugs [14].

However, no data on the efficacy and safety of IND as an adjuvant for epidural labor analgesia are available. Our hypothesis was that pretreatment with a single low dose (0.5 μg/kg) of IND 15 min before an epidural puncture would improve labor analgesia with a faster onset and less procedural pain. We tested this hypothesis in a prospective, randomized, double-blind, controlled study of nulliparous patients.

## Methods

### Research and design

This is a prospective, randomized, double-blind controlled trial approved by the Institutional Ethics Committee of Anhui Women and Child Health Care Hospital (No. 2017(10)) in accordance with the Declaration of Helsinki, and written informed consent was obtained from all participants in the trial. The study was registered at Chictr.org.cn on 29/05/2020 with the registration number ChiCTR2000033356 (http://www.chictr.org.cn/listbycreater.aspx). This manuscript adheres to the applicable CONSORT guidelines.

### Participants

From June to December 2020, healthy nulliparous women at 37 to 42 gestational weeks in spontaneous labor and desiring epidural labor analgesia at Anhui Women and Child Health Care Hospital were eligible. Inclusion in this study was based on the following criteria [8]: (1) maternal age ranging from 20 to 35 years; (2) American Society of Anesthesiologists (ASA) class I-II; (3) body mass index (BMI) 20–35 kg/cm^2^; (4) singleton and spontaneous labor with cervical dilatation 2–3 cm; and (5) spontaneous labor pain with a visual analogue scale (VAS) score > 5 cm (VAS scores: 0 = no pain, 10 = worst pain imaginable). The exclusion criteria were as follows [8, 15]: (1) parturients refused to participate; (2) ASA class III-IV; (3) parturients complicated with severe cardiopulmonary or brain dysfunction; (4) parturients with HR < 50 bpm; (5) parturients had a history of opioid abuse; (6) parturients suffered from severe mental disorders and could not cooperate with the anesthesiologist; (7) parturients with BMI < 20 or > 35 kg/cm^2^ because they often require an appropriately adjusted administration scheme for epidural labor analgesia; (8) cervical dilatation > 3 cm; (9) parturients with multiple deliveries or preterm deliveries (< 37 weeks); (10) any contraindications to epidural puncture, such as spinal deformity or coagulation dysfunction; and (11) those requiring cesarean section or induction of labor or delivery within 1 h after epidural catheterization. None of the parturients received any other analgesic treatment before the implementation of labor analgesia.

### Randomization and concealment of group

Parturients were randomly divided into the conventional PIEB group (CON, n = 34) or the intranasal dexmedetomidine before PIEB group (IND, n = 33) through a computer-generated random number sequence using SPSS version 22.0 (IBM Corp, USA). To maintain the blindness of the study, two anesthesia personnel were involved. The anesthesiologist prepared the drug and placebo and administered them 15 min before the analgesia procedure. The anesthesia nurse was responsible for the management of labor analgesia and the assessment and recording of the data. The parturients and the outcome assessor (the anesthesia nurse) were blinded to the group allocations. A senior anesthesiologist-in-charge who had previously completed over 1000 neuraxial punctures conducted the analgesia procedures.

### Epidural analgesia procedure

Upper arm venous access was established via a 22G intravenous indwelling needle. Then, parturients were brought into the labor analgesia room for the epidural analgesia procedure. Noninvasive blood pressure (NIBP), heart rate (HR) and pulse oximetry (SpO_2_) were monitored at 10-min intervals. The intensity of uterine contractions (IUCs) and fetal heart rate (FHR) were monitored continuously using an external tocodynamometry (TOCO) system.

In the IND group, 100 μg/ml dexmedetomidine hydrochloride (You Bi Tuo; Jiangsu Yang Zi Jang Medicine Co., Ltd.) was prepared without further dilution [16], 0.5 μg/kg of which was administered by the anesthesiologist through a bilateral nasal drip using a 1 ml syringe 15 min before the epidural puncture. An equal volume of normal saline was given in the same manner in the CON group.

Epidural puncture was performed at the L2-3 interspace with an 18-gauge epidural needle via the loss-of-resistance-to-saline method. Briefly, parturients were placed in the left decubitus and knee-hugging position, and 2% lidocaine was administered as local anaesthesia. A guide needle was used to penetrate the skin and supraspinous ligament. The epidural needle pierced the skin, supraspinous ligament, and interspinous ligament along the guiding pinhole and was then slowly advanced. When the tip passed through the ligamentum flavum, the loss of resistance to saline or the appearance of negative pressure indicated that the tip entered the epidural space. When no outflow of cerebrospinal fluid or blood return was observed by syringe suction, a small amount of saline was injected without resistance, which proved that the puncture was successful.

An epidural catheter was then inserted 4.5 cm cephalad into the epidural space. Five minutes after a test dose of 5 ml of 1% lidocaine, labor analgesia was initiated with 10 mL of 0.083% ropivacaine (AstraZeneca, Sweden) combined with 0.4 μg/mL sufentanil (Yichang HumanWell, China). Seven millilitres of this mixed solution was administered every 30 min for maintenance of labor analgesia with a programmed intermittent epidural bolus (PIEB). The PCEA dose was 7 ml with a lockout time of 30 min.

## Data collection

### Demographic and baseline characteristics

Data were recorded by investigators who were blinded to the purpose of the study. Maternal age, BMI, ASA class, gestational weeks, initial VAS score, patients on oxytocin infusion at the time of epidural placement and cervical dilation at the time of epidural administration were recorded. Maternal SpO_2_, systolic blood pressure (SBP), HR and fetal heart rate (FHR) were monitored and recorded during the interval between two contractions as baseline characteristics.

### Primary outcome assessment

The primary outcomes of this study were the onset time of analgesia and the procedural pain of the epidural puncture. The onset of analgesia was defined as the time from the start of the epidural injection to when the VAS scores ≤ 3 during active uterine contractions [2]. The pain felt during the epidural puncture was evaluated with VAS scores during the procedure [15].

### Secondary outcome assessments

Secondary outcomes included the following: VAS pain score, Ramsay Sedation Score (RSS), FHR and intensity of uterine contractions (IUCs) during the first 60 min after epidural block (T0: baseline, T1: 5 min after block, T2: 10 min after block, T3: 15 min after block, T4: 30 min after block, T5: 60 min after block). Consumption of analgesic drugs in the PIEB pump, PCEA boluses, delivery mode, duration of labor, neonatal Apgar scores, maternal satisfaction and adverse effects were assessed within two hours of delivery. The efficacy of epidural analgesia was assessed using the visual analogue scale. The intensity of uterine contractions and fetal heart rate were monitored and recorded using an external tocodynamometry (TOCO) system (SRF618B5, Sunary Company, Guangdong, China) [17]. “Adequate analgesia” was defined as a VAS score ≤ 3 during active uterine contractions [2]. Failure to reach the onset of analgesia was defined as cases that failed to reach adequate analgesia by 15 min after the initial dosage. The RSS scoring criteria were as follows [8]: 1, anxious and restless; 2, cooperative, oriented and quiet; 3, responsive to commands; 4, asleep but quickly responded to a light glabellar tap or loud auditory stimulus; 5, asleep, sluggish response to a light glabellar tap or loud auditory stimulus; and 6, asleep, no response. Excessive sedation was defined as RSS score > 4 [8]. Adverse effects included hypotension, maternal or fetal bradycardia, excessive sedation, nausea/vomiting, pruritus, shivering and respiratory depression. Respiratory depression was defined as SpO_2_ < 90% when inhaling air and nasal oxygen inhalation was then administered at a rate of 2–3 L/min. Hypotension was defined as a systolic blood pressure < 90 mmHg or a decrease > 20% from baseline (before analgesia) and treated with positioning into a left supine position or IV boluses of 0.5 mg metaraminol [18]. Maternal bradycardia was defined as a decrease in HR < 50 bpm and treatment with IV boluses of 0.25 mg atropine. Fetal bradycardia was defined as a FHR < 120 bpm and duration of > 10 min and was treated by an obstetrician according to the department’s clinical guidelines. Maternal satisfaction with labor analgesia was assessed using a VRS (0 very unsatisfied, 10 very satisfied) [19, 20].

## Statistical analysis

### Sample size calculation

To calculate the sample size, a pilot study was performed to evaluate the time to adequate analgesia (VAS ≤ 3) in 10 patients, with 5 patients receiving normal saline intranasally (CON) and 5 receiving 0.5 μg/kg dexmedetomidine hydrochloride intranasally (IND) 15 min before the epidural procedure. The median onset time of the CON group was 10.2 min, while that of the IND group was 7.9 min. The standard deviations (SDs) were 2.1 and 2.6, respectively. For a power of 90% and two-sided statistical significance set at 0.05, the minimum sample size was calculated by PASS15.0 software to be 24 patients per group [21]. Given an expected patient dropout rate of 20%, the total sample size was increased to 60 participants (30 patients per group).

### Primary outcome analysis

The median time to achieving adequate epidural analgesia was estimated using Kaplan–Meier survival curves [22]. A 95% confidence interval (CI) around the median was reported and compared between groups via the log-rank test. A univariate Cox regression model was used to evaluate the relative risk of achieving adequate pain control in the different groups. VAS scores during epidural puncture did not have a normal distribution and therefore are expressed as the median (95% CIs) and were examined using the Mann–Whitney *U* test.

### Secondary outcome analysis

The one-sample Kolmogorov–Smirnov test was used to assess the normality of the quantitative data. Normally distributed output data are presented as the mean (standard deviation, SD) and were compared using Student's *t* test, such as umbilical artery blood gas analysis. Skewed data, such as the number of PCEA boluses and maternal satisfaction, were summarized as the median (95% CIs) and compared between groups using the Mann–Whitney *U* test. Categorical variables, such as the incidence of adverse effects and failed onset of analgesia, were reported as frequencies (percentages) and compared using the chi-squared (χ^2^) test.

VAS, RSS, IUC and FHR were assessed longitudinally between groups with a linear mixed model using the restricted maximum likelihood method and accounting for patient-level clustering (random intercept) under an unstructured model. The baseline values of VAS, RSS, IUC and FHR (T0) were included as covariates to ensure statistical balance and reduce error variance [23]. The models consisted of the main effects for the treatment group and time. The group-by-time interaction term was tested first. If significant, between-group differences at each time point were tested with adjusted Bonferroni correction for multiple comparisons. If not significant, the treatment main effect was tested next. *P* < 0.05 was considered statistically significant. All statistical analyses were performed using SPSS version 22.0 (IBM Corp, USA). This trial was prospectively registered at Chictr.org.cn on 29/05/2020 with the registration number ChiCTR2000033356 (http://www.chictr.org.cn/listbycreater. aspx).

## Results

**Fig. 1:**
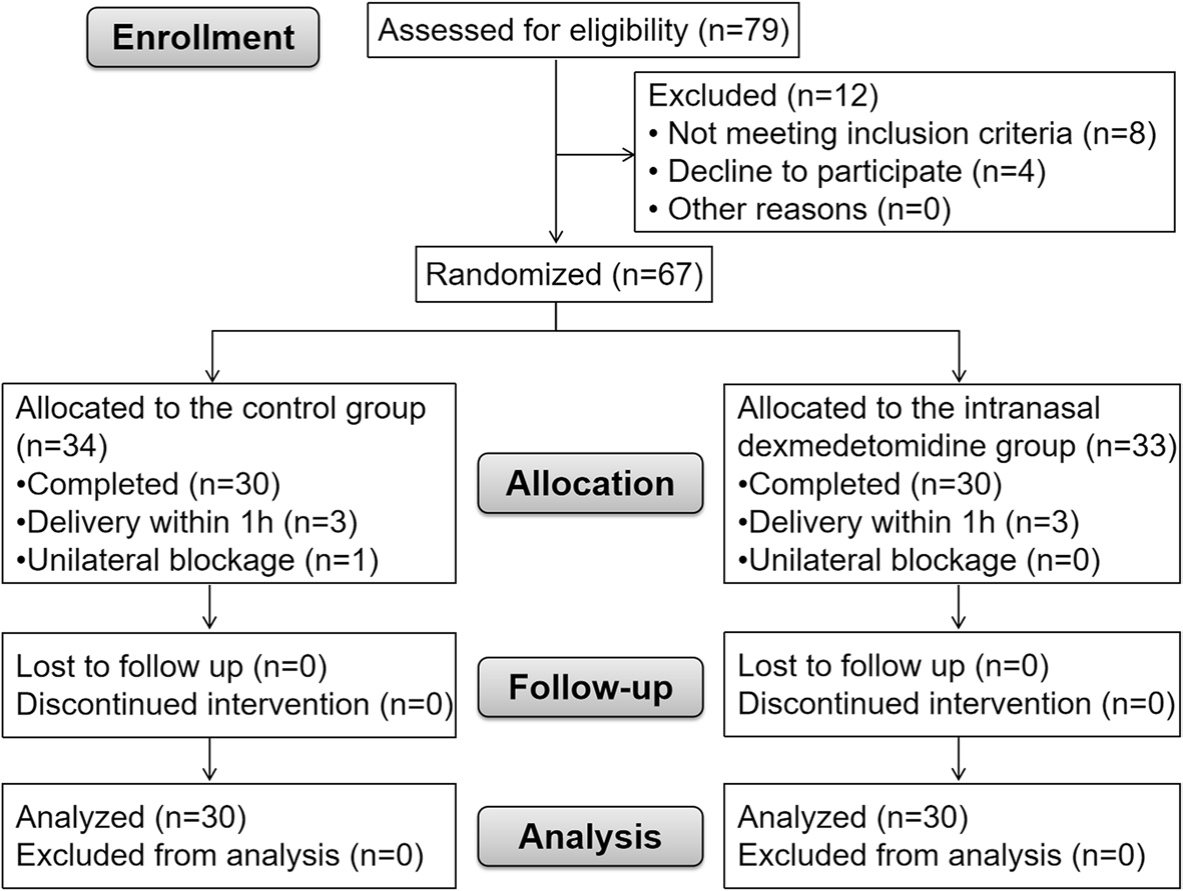
CONSORT trials flow diagram. The control group (CON), the conventional PIEB group; The intranasal dexmedetomidine group (IND), the PIEB with intranasal dexmedetomidine group; PIEB, programmed intermittent epidural bolus. CONSORT, Consolidated Standards of Reporting Trials

From June to December 2020, 79 women were screened, and 67 subjects were recruited and randomized into two groups. Seven participants were excluded due to delivery within one hour or unilateral blockage, and data were collected from 60 subjects (Fig. 1). Demographic and baseline characteristics, including age, ASA class, BMI, gestational weeks, initial VAS score, patients on oxytocin infusion at the time of epidural placement, cervical dilatation at the time of epidural block as well as baseline maternal SpO2, SBP, HR, FHR were comparable between the two groups (*P* > 0.05, Table 1).

**Table 1:** Demographic and baseline characteristics of parturients

	**CON (*n* = 30)**	**IND (*n* = 30)**	***Z/t/x***^***2***^	***P* value**
Age (y)	27.9 ± 2.8	27.3 ± 2.9	0.860	0.393
BMI (kg/m^2^)	25.6 ± 3.0	25.7 ± 3.0	0.112	0.911
Gestational age (w)	39.8 ± 0.9	39.6 ± 1.0	0.972	0.335
Cervical dilatation at the time of epidural block (cm)	3.0 (2.6–2.9)	3.0 (2.6–2.9)	0.605	0.545
Patients on oxytocin infusion at time of epidural placement	18 (60.0)	22 (73.3)	1.200	0.273
Initial VAS score	9.0 (8.5–9.3)	9.0 (8.7–9.4)	-0.522	0.602
Baseline SBP (mmHg)	121.6 ± 6.4	123.0 ± 7.3	0.826	0.413
Baseline SpO2 (%)	99.5 (99.0–100.0)	99.0 (98.8–100.0)	-0.139	0.164
Baseline maternal HR (bpm)	94.8 ± 9.0	93.80 ± 7.9	0.457	0.649
Baseline FHR (bpm)	143.5 (142.7–146.2)	145.0 (142.2–145.7)	-0.473	0.636
ASA physical status			0.000	1.000
Class 1	27 (90.0)	26 (86.7)		
Class 2	3 (10.0)	4 (13.3)		

Values are presented as mean ± SD, n (%) or median (95% CI) depending on the distribution of the sample. *P* < 0.05 is set to be significant. CON, the conventional PIEB group; IND, the PIEB with intranasal dexmedetomidine group; PIEB, programmed intermittent epidural bolus. VAS, Visual analog scale; SBP, Systolic blood pressure; SpO_2_, Pulse oximetry; HR, Heart rate; FHR, Fetal heart rate; VAS, Visual analog scale; BMI, Body mass index

### Primary outcomes

**Fig. 2:**
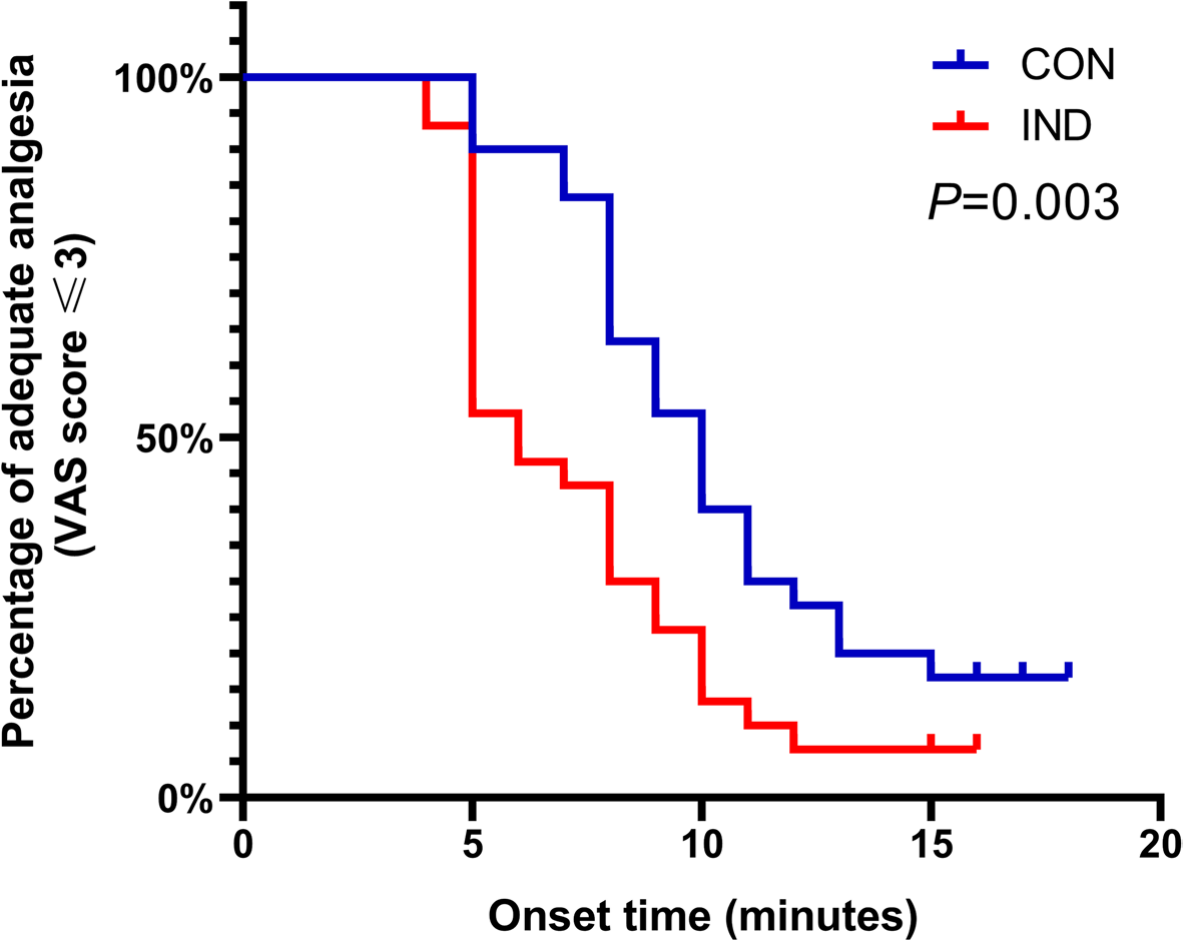
Kaplan–Meier curves for time to achieving adequate analgesia after loading dose. The difference between groups was significant, *P* = 0.003. According to the univariate Cox regression analysis, parturients in the IND group had a significantly faster onset of achieving VAS pain score ≤ 3 than that in the CON group (hazard ratio (HR) = 2.069; 95% CI, 2.187–3.606; *P* = 0.010). The VAS scores were recorded at every time of active uterine contractions and the onset time was recorded as the time from beginning of epidural block to achieving adequate analgesia. “Adequate analgesia” was defined as VAS score ≤ 3 during active uterine contractions. CON, the conventional PIEB group; IND, the PIEB with intranasal dexmedetomidine group; PIEB, programmed intermittent epidural bolus

The primary outcomes are shown in Fig. 2 and Table 2. According to the univariate Cox regression analysis, parturients in the IND group had a significantly achieving VAS pain scores ≤ 3 than those in the CON group (hazard ratio (HR) = 2.069; 95% CI, 2.187–3.606; *P* = 0.010). The median time (95% CIs) until adequate analgesia was 10.0 (8.5 to 11.5) minutes in CON and 6.0 (4.9 to 7.1) in IND (*P* = 0.003 via log-rank test, Table 2). The VAS scores during the epidural puncture in the IND group were also significantly lower than those in the CON group (2.0 (1.8–2.5) *vs.* 3.5 (3.3–4.5), *P* ≤ 0.001, Table 2).

**Table 2:** Characteristics of analgesia, labor and newborn outcome

	CON (*n* = 30)	IND (*n* = 30)	*Z/t/x*^*2*^	*P* value
Onset of analgesia (min)	10.0 (8.5–11.5)	6.0 (4.9–7.1)	8.532	0.003
VAS during epidural puncture	3.5 (3.3–4.5)	2.0 (1.8–2.5)	-4.892	0.000
Consumption of ropivacaine (mg)	54.8 ± 24.4	40.9 ± 25.4	2.153	0.036
Consumption of sufentanil (μg)	27.4 ± 12.2	20.5 ± 12.7	2.153	0.036
Number of total PCEA boluses (n)	2.0 (1.3–2.6)	1.0 (0.6–1.3)	-2.540	0.011
Maternal satisfaction (n, %)	9.0 (8.2–8.9)	9.0 (9.1–9.5)	-3.261	0.001
Failed to reach onset of analgesia (n, %)	6 (20.0)	2 (6.7)	1.298	0.255
Duration of first stage (min)	420.0 (397.1–577.8)	360.0 (365.3–541.9)	-0.632	0.528
Duration of second stage (min)	37.0 (32.7–55.4)	39.0 (34.0–61.8)	-0.240	0.811
Mode of delivery (n, %)			0.000	1.000
Vaginal	29 (96.7)	28 (93.3)		
Cesarean delivery	1 (3.3)	2 (6.7)		
Postpartum bleeding (ml)	150 (139.9–445.5)	150 (157.3–207.4)	-0.456	0.649
Neonatal Apgar score				
1 min	9.0 (8.8–9.1)	9.0 (8.8–9.1)	-0.023	0.982
5 min	10.0 (9.8–10.0)	10.0 (9.8–10.0)	-0.463	0.643
Umbilical artery PH	7.3 ± 0.1	7.3 ± 0.0	-0.533	0.596
Umbilical artery PaO_2_ (mmHg)	32.6 ± 4.3	33.7 ± 4.1	-0.994	0.324
Umbilical artery Lac (mmol/L)	2.7 ± 0.6	2.6 ± 0.7	0.381	0.705

Values are presented as mean ± SD, n (%) or median (95% CI) depending on the distribution of the sample. *P* < 0.05 is set to be significant. CON, the conventional PIEB group; IND, the PIEB with intranasal dexmedetomidine group; PIEB, programmed intermittent epidural bolus. VAS, Visual analog scale; PCEA, Patient-controlled epidural analgesia; PH, Pondus Hydrogenii; Lac, Lactic acid level; PaO2, Partial arterial oxygen pressure

### Secondary outcomes

**Fig. 3:**
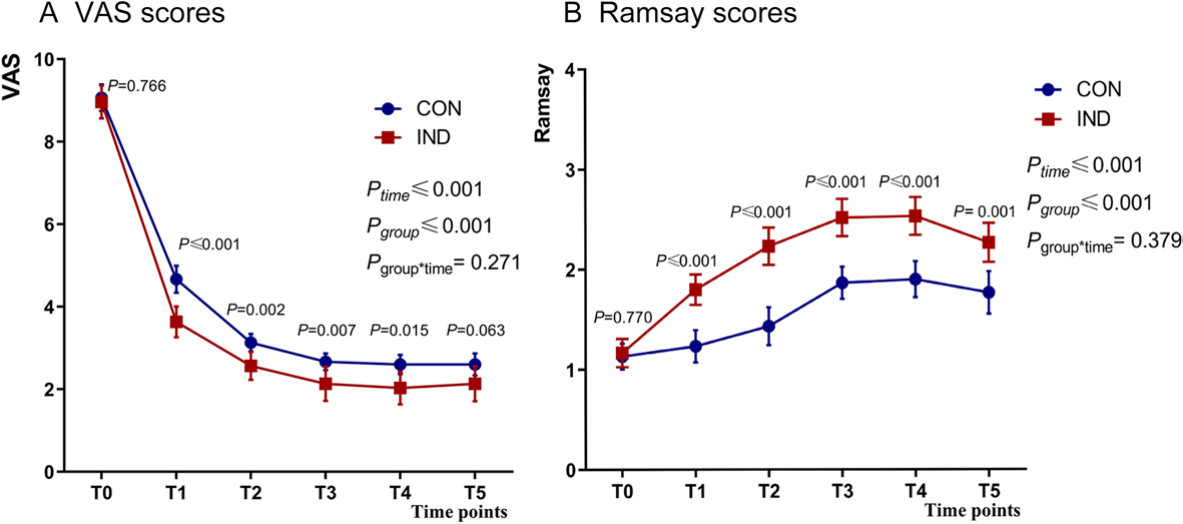
**A** Mean VASs during the first 60 min after epidural block. **B** Mean RSS values during the first 60 min after epidural block. CON, the conventional PIEB group; IND, the PIEB with intranasal dexmedetomidine group; PIEB, programmed intermittent epidural bolus. VAS, Visual analog scale; RSS, Ramsay Sedation Score. Data are shown as the appearance of mean and error and the plot of 95% CI and analyzed via the liner mixed model and adjusted Bonferroni correction for multiple comparisons. A *P*-value less than 0.05 was considered to be statistically significant. Values at T0 were included as covariate for baseline correction. T0: before block, T1: 5 min after block, T2: 10 min after block, T3: 15 min after block, T4: 30 min after block, T5: 60 min after block

The linear mixed-effect model showed that the intergroup effect over time between the CON and IND groups of VAS and RSS scores had significant differences (F = 17.180, *P* ≤ 0.001, mean difference [95% CI], 0.582 [0.301 to 0.864] and F = 45.823, *P* ≤ 0.001, mean difference [95% CI], 0603 [0.424 to 0.781], respectively), Fig. 3A and B. When examining the group-by-time interaction, there were no significant differences during the first hour after initiating epidural analgesia in the VAS and RSS scores, Fig. 3A and B. According to the results by adjusted Bonferroni correction for multiple comparisons at each time point, the VAS scores significantly decreased during the first 30 min post-analgesia (*P* = 0.766, *P* < 0.001, *P* = 0.002, *P* = 0.007, *P* = 0.015, *P* = 0.063, Fig. 3A), and the RSSs significantly increased in the first 30 min post-analgesia (*P* = 0.770*, P* < 0.001, *P* < 0.001, *P* < 0.001, *P* < 0.001, *P* = 0.001, Fig. 3B) in the IND group compared with the CON group.

**Fig. 4:**
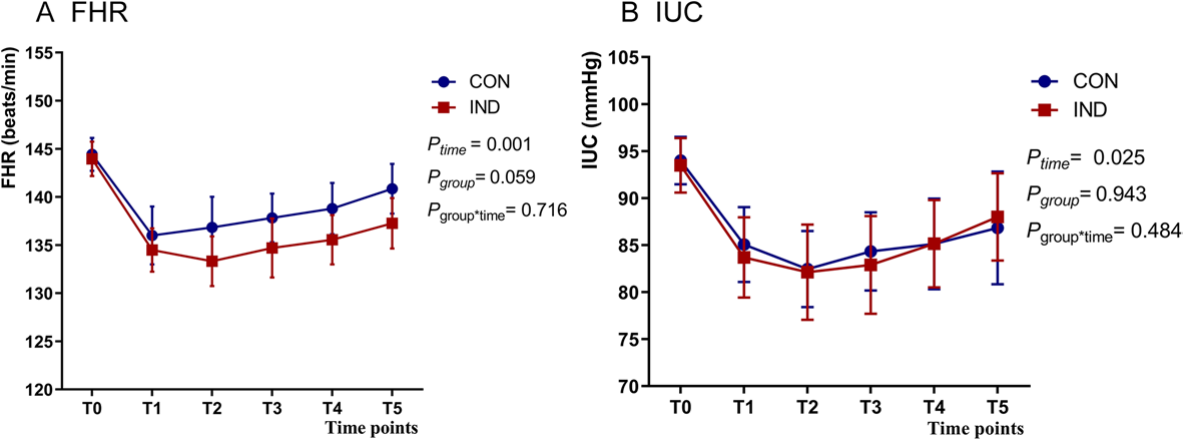
**A** The FHR during the first 60 min after epidural block. **B** The intensity of IUC during the first 60 min after epidural block. CON, the conventional PIEB group; IND, the PIEB with intranasal dexmedetomidine group; PIEB, programmed intermittent epidural bolus. FHR, Fetal heart rate; IUC, intensity of uterine contraction. Data are shown as the appearance of mean and error and the plot of 95% CI and analyzed via the liner mixed model, and adjusted Bonferroni correction for multiple comparisons. A *P*-value less than 0.05 was considered to be statistically significant. Values at T0 were included as covariate for baseline correction. T0: before block, T1: 5 min after block, T2: 10 min after block, T3: 15 min after block, T4: 30 min after block, T5: 60 min after block

The FHR and IUC data were also analysed via a linear mixed-effect model. The intergroup effect over time between the CON and IND groups of the FHR and IUC had no significant difference (F = 3.702, *P* = 0.059, mean difference [95% CI], 2.993 [-0.121 to 6.108] and F = 0.005, *P* = 0.943, mean difference [95% CI], 0.213 [-5.715 to 6.142], respectively), Fig. 4A and B. When examining the effect of time and the group-by-time interaction of the FHR and IUC scores, there were also no significant differences during the 60 min after initiating epidural analgesia (Fig. 4A and B). When the two groups of data were analysed separately, the time effect of FHR in the CON and IND groups was significantly different (F = 4.658, *P* = 0.002, 95% CI [135.7 to 140.4 beats/minutes] and F = 3.494, *P* = 0.011, 95% CI [132.9 to 137.2 beats/minutes], respectively), with a temporary decrease during the first 5 and 10 min, respectively. The time effect of IUC had no significant difference in the CON group (F = 0.371, *P* = 0.829, 95% CI [80.1 to 88.8 mmHg]) but had a significant difference in the IND group (F = 3.606, *P* = 0.009, 95% CI [80.1 to 88.5 mmHg]), with a temporary decrease during the first 10 min and then gradual recovery (Fig. 4A and B).

In the IND group, the consumption of analgesics and the number of PCEA boluses were significantly lower than those in the CON group (*P* = 0.036*, P* = 0.011), and maternal satisfaction was significantly higher than that in the CON group (*P* = 0.001) (Table 2). Six patients in the CON group (20.0%) and two in the IND group (6.7%) did not achieve VAS scores ≤ 3 in the 15-min study period, and there was no significant difference between the two groups (*P* = 0.255). Delivery mode, duration of the first and second stage, neonatal Apgar scores, umbilical artery blood gas analysis and the occurrence of adverse effects were comparable between the two groups (*P* > 0.05, Tables 2 and 3).

**Table 3:** Adverse effects of epidural analgesia

	CON (*n* = 30)	IND (*n* = 30)	*Z/t/* × *2*	*P* value
Hypotension	0 (0.0)	0 (0.0)	0.000	1.000
Respiratory depression	0 (0.0)	0 (0.0)	0.000	1.000
Maternal Bradycardia	0 (0.0)	1 (3.3)	0.000	1.000
Fetal bradycardia within 30 min after EA	2 (6.7)	3 (10.0)	0.000	1.000
Excessive sedation	0 (0.0)	0 (0.0)	0.000	1.000
Nausea and vomiting	3 (10.0)	1 (3.3)	0.268	0.605
Pruritus	2 (6.7)	0 (0.0)	0.000	1.000
Shivering	3 (10.0)	0 (0.0)	1.404	0.236

Values are presented as n (%). *P* < 0.05 is set to be significant. CON, the conventional PIEB group; IND, the PIEB with intranasal dexmedetomidine group; PIEB, programmed intermittent epidural bolus; EA, epidural analgesia

## Discussion

The key findings of our study were that pretreatment with low-dose intranasal dexmedetomidine (IND) as an adjunct with the initiation of epidural labor analgesia provided a quicker onset of analgesia and lower pain scores caused by epidural puncture than conventional epidural labor analgesia (CON).

A number of studies have shown that administering DEX intranasally is safe, effective, more comfortable and convenient than an intravenous approach [16]. To the best of our knowledge, no study has investigated the efficacy of IND as an adjuvant for epidural labor analgesia to date. However, intravenous DEX was reported as an adjunct for labor analgesia and cesarean anesthesia in a parturient with a tethered spinal cord [6] and for labor analgesia in a parturient with preeclampsia [24]. The results showed that intravenous DEX improved analgesia and sedation without episodes of maternal hypotension, bradycardia or fetal heart rate distress [6, 24]. Uemura et al. used a clinically relevant dosing regimen (a bolus injection of dexmedetomidine 1.0 μg/kg) on pregnant ewes and suggested that intravenous DEX produces significant maternal sedation without altering the fetal physiologic status [7]. In addition, in vitro studies on human placentas have demonstrated low maternal fetal transfer of DEX, presumably due to its high lipophilicity [25]. These data suggest that intravenous DEX as an adjunct for labor analgesia is safe for the mother and fetus.

The nasal mucosa is rich in capillaries, and intranasal DEX administration results in the rapid entry of the drug into the bloodstream [13]. Compared to the intravenous route, IND has the same metabolic pathway but causes a more gradual ascending peak plasma concentration and hence better tolerability and convenience [26]. Previous studies found that IND was an easy and noninvasive route with a high bioavailability of 81.8% and more than 84% in pediatric patients compared with the intramuscular and oral routes [27]. Iirola et al. reported that IND had a bioavailability of 65% (35–93%) in healthy men [28].

This research showed that the median onset time of traditional PIEB with ropivacaine and sufentanil was 10.0 (8.5 to 11.5) minutes, consistent with the results of Song et al. [3]. The median onset time of IND combined with PIEB was 6.0 (4.9 to 7.1) minutes. DEX has sedative and analgesic properties through the activation of α2-adrenoreceptors and has been widely reported as an anesthesia adjunct to the intravenous and epidural routes to improve the effect of labor analgesia, reduce the total consumption of analgesics and lengthen the duration of analgesia without shortening the onset time [6, 8, 9, 29]. Li et al. [26] reported that the onset time of IND by atomizer (47.5 min, 95% CI, 25 to 135 min) and by drops (60 min, 95% CI, 30 to 75 min) was significantly longer than intravenous DEX (15 min, 95% CI, 15 to 20 min). However, the reported onset times of IND were inconsistent and ranged from 7 to 31 min in a systematic review reported by Poonai et al. [30]. According to the above literature, we designed the pretreatment time of IND as 15 min before the epidural puncture plus the time of the epidural puncture operation, which required 15–20 min. This study showed that the median onset time of epidural analgesia in the IND group was 6.0 min, which might be because we preadministered IND 15 min before the epidural puncture, providing preemptive and auxiliary analgesia. A recent study also reported that a combination of 0.1% ropivacaine with 0.25 μg/ml Dex + 0.25 μg/ml sufentanil yielded a shorter onset time than that of 0.1% ropivacaine + 0.5 μg/ml Dex or 0.5 μg/ml sufentanil in epidural labor analgesia [29]. Preemptive analgesia is an intervention prior to initiating painful stimuli by blocking the establishment of altered central processing of afferent input, which inhibits hyperalgesia and elevates the pain threshold after surgery to reduce or prevent subsequent pain [31]. The present study found that premedication with IND decreased procedural distress with greater compliance for the epidural puncture than traditional PIEB (CON) with preemptive analgesia and sedation. Poonai et al. reported that IND was likely more effective for procedural distress in children than oral chloral hydrate and oral midazolam [30]. Many parturients in labor are also anxious due to uterine contractile pain and the distress of the epidural puncture. DEX relieves anxiety and reduces plasma levels of stress hormones [32, 33]. In addition, it is necessary for the parturient to be able to cooperate with the health care staff while relieving pain. YUAN et al.’s study showed that IND combined with local anesthesia resulted in conscious analgesia, sedation and improved anesthetic effects during breast lumpectomy [16]. Here, IND was used to relieve maternal anxiety and did not affect cooperation during delivery. The RSSs of all parturients were less than 3 in this study, with no respiratory depression or reflux aspiration. Farghaly et al. [34] reported that DEX increased the pain threshold of nerve cells, which might be one of the mechanisms responsible for relieving procedural pain. Therefore, IND might alleviate procedural pain through preemptive analgesia, elevating the pain threshold and providing superior sedation.

The main purpose of this study was to observe the initiation of labor analgesia; therefore, the data were only collected during the first 60 min of this study. VAS scores were decreased in the IND group in the first 30 min, but no significant difference was seen 60 min post-analgesia, which suggested that IND improved the initiation of analgesia without affecting the maintenance of analgesia. A higher RSS was found in the IND group, which suggested that IND might reduce the stress and anxiety of parturients during labor. DEX has also been confirmed to improve sedation during the later stages of labor via epidural administration [8, 9]. YUAN et al. demonstrated that IND provided good clinical analgesia and sedation for at least 70 min after administration [16].

In this study, the median difference in the onset of analgesia between the CON and IND groups was 4 min. Wang et al. assumed that any difference should be at least 5 min to be clinically significant [2]. However, in our clinical practice, parturients who suffer from uterine contraction distress are eager to alleviate the pain as soon as possible. Hence, a median difference of 4 min was considered clinically meaningful, which was also supported by a recent study by Wang et al. [15]. In addition, although the effect of labor analgesia was significantly different between the two groups, the anesthesiologists took measures to meet the analgesic needs of parturients with the addition of analgesic drugs as necessary. Therefore, the maternal satisfaction score was as high as 9 in both groups. In the IND group, a lower bolus frequency of PCEA or physician rescue analgesia and less consumption of analgesic drugs were needed to achieve a high maternal satisfaction score, which suggested that pretreatment with IND as an adjunct of PIEB also improved the quality of labor analgesia maintenance.

DEX has a biphasic effect on SBP, as it decreases the heart rate and cardiac output via central anti-sympathetic action but increases vascular resistance via peripheral vascular α1-adrenoreceptor activation. Therefore, SBP is decreased and then elevated with increasing plasma concentrations in humans [35]. Li et al. considered that IND was associated with a longer onset than intravenous access. A more gradual onset avoided the α1-adrenoreceptor agonist effects seen with rapid intravenous access (hypertension and bradycardia) [26]. This study found that adverse effects such as maternal bradycardia, hypertension and hypotension were absent in both groups. There was no significant difference in FHR between the two groups, but there was a temporary decrease 5–10 min after the loading dose. Transient fetal bradycardia presented in 2 and 3 cases in the CON and IND groups, respectively, which might be associated with the loading dose of sufentanil [36]. No significant differences were detected in delivery mode, first and second stage duration, neonatal Apgar scores, umbilical blood gas analysis or side effects between the two groups, consistent with previous studies [2, 9].

Excessive uterine contractions might induce fetal distress or placental abruption. Several studies have suggested that DEX enhances uterine constriction [37, 38], and the potential detriment of changes in uterine contractions should be considered. The current literature about the impact of DEX on uterine contraction is inconsistent. As shown by Kimizuka et al. via in vitro studies, DEX presented a dose-dependent enhancement of myometrial spontaneous contraction in humans and rats without increasing oxytocin-induced uterine contractions by increasing the sensitivity of muscle fibres to calcium ions [38]. However, DEX was also confirmed to enhance oxytocin-induced myometrium contractions by in vivo studies [38]. In another in vitro study, Öcal et al. showed that DEX caused an increase in spontaneous contraction forces and frequency in early and middle pregnancy in rats but had the opposite effects in late pregnancy in a dose-dependent manner [37]. An in vitro study by Gertler et al. suggested that clinically relevant concentrations of DEX reduced uterine contractility by cell membrane hyperactivation and reducing the influx of ATP, norepinephrine, and calcium ions via a negative feedback mechanism [39]. Epidural analgesia was confirmed to inhibit uterine contraction by decreasing the secretion of endogenous oxytocin [40, 41], which could also counteract the effect of DEX on uterine contractions. In this study, we observed that the uterine contraction intensity in the IND group decreased briefly during the first 10 min and then recovered gradually, and there was no significant difference compared to the CON group. No episodes of fetal distress or placental abruption induced by excessive uterine contractions occurred, and no significant differences in delivery mode or first- and second-stage duration were detected. In summary, using a clinically appropriate concentration and dosage of DEX and epidural local anaesthetics are of paramount importance to avoid adverse effects on uterine contractions.

To identify the optimal intranasal dose for providing the best analgesia and sedation while minimizing side effects, three different intranasal doses (0.5, 0.8 and 1.0 μg/kg) were tested in a preliminary experiment according to methods used in previous studies. We found that dosages of 0.8 and 1.0 μg/kg reduced uterine contractions and extended the first stage of labor. This result was inconsistent with a previous study [16]. The first reason for this might be that most of the subjects were children, who have different pharmacokinetics and apparent volumes of distribution than adults. In addition, the optimal dose of IND is often related to the analgesic intensity produced by the subject drug and the degree of surgical trauma. In this study, PIEB played the major role in analgesia, and IND was used as an adjunct to improve analgesia and sedation. Uusalo et al. also demonstrated that intraoperative use of low-dose IND (0.5 μg/kg) yielded improved clinical sedation and analgesia and reduced opioid consumption in adults undergoing total hip arthroplasty (THA) [5].

This study had several limitations. First, the study was a single-centre study, and the sample was representative but not large. Second, the optimal dosage was not selected using a sequential method, which may reduce the power of the study. Third, the population was young women (27.9 ± 2.8 and 27.3 ± 2.9), which accounts for the majority of current primipara undergoing vaginal delivery, while the population of “older mothers” has increased in recent years and should be further studied. Finally, only data from 60 min post-IND were collected to assess the effect of IND on onset time and procedural pain. Whether IND has advantages in the maintenance of analgesia and breakthrough pain should be explored in future studies.

In summary, pretreatment with intranasal low-dose dexmedetomidine as an adjunct to a programmed intermittent epidural bolus might provide faster analgesia onset and less pain during the epidural puncture without increasing adverse effects compared to the traditional PIEB mode. These findings suggest that pretreatment with IND could be a useful adjunct for some patients during the initiation of epidural analgesia, and further investigation should be encouraged to determine its utility more fully.

## Data Availability

The data are available from the corresponding author if there is a later request.

## Abbreviations

EA: Epidural analgesia
PIEB: Programmed intermittent epidural bolus
CEI: Continuous epidural infusion
DPE: Dural puncture epidural
DEX: Dexmedetomidine
IND: Intranasal dexmedetomidine
ASA: American Society of Anesthesiologists
SpO2: Pulse oximetry
FHR: Fetal heart rate
IUC: Intensity of uterine contraction
NIBP: Non-invasive blood pressure
SBP: Systolic blood pressure
HR: Heart rate
CI: 95% Confidence interval
SD: Standard deviation
BMI: Body mass index
PCEA: Patient-controlled epidural analgesia
VAS: Visual analog scale
RSS: Ramsay Sedation Score
PH: Pondus Hydrogenii
Lac: Lactic acid level
PaO2: Partial arterial oxygen pressure
